# Vitamin D3 Supplementation Modulates Inflammatory Protein CHI3L1/YKL‐40 and Oxidative Stress Status in Multiple Sclerosis

**DOI:** 10.1002/npr2.70076

**Published:** 2025-11-23

**Authors:** Sevda Asadpour, Mehrdokht Mazdeh, Jamshid Karimi, Iraj Khodadadi, Gholamreza Shafiee

**Affiliations:** ^1^ Department of Clinical Biochemistry, Medicine School Hamadan University of Medical Sciences Hamadan Iran; ^2^ Department of Neurology School of Medicine, Hearing Disorder Research Center, Avicenna Institute of Clinical Sciences, Sina (Farshchian) Educational and Medical Center, Hamadan University of Medical Sciences Hamadan Iran; ^3^ Department of Clinical Biochemistry School of Medicine, Nutrition Health Research Center, Institute of Health Sciences and Technology, Hamadan University of Medical Sciences Hamadan Iran; ^4^ Department of Clinical Biochemistry Medicine School, Nutrition Health Research Center, Hamadan University of Medical Sciences Hamadan Iran

**Keywords:** antioxidant enzymes, CHI3L1 (YKL‐40), multiple sclerosis, oxidative stress, vitamin D3

## Abstract

**Objective:**

Multiple sclerosis (MS) is characterized by chronic neuroinflammation and oxidative stress. Vitamin D is believed to exert immunomodulatory and antioxidant effects, yet its impact on specific inflammatory proteins such as CHI3L1 (YKL‐40) in MS remains unclear. This study evaluated whether 8‐week vitamin D3 supplementation affects serum CHI3L1 levels, oxidative stress markers, and antioxidant enzyme activities in patients with MS.

**Methods:**

In this single‐arm pre‐post clinical trial, 35 patients with MS (aged 30–56 years) received oral vitamin D3 supplementation (50 000 IU/week) for 8 weeks. Serum 25(OH)D and CHI3L1 levels were determined using commercial enzyme‐linked immunosorbent assay (ELISA) kits. oxidative stress markers were measured pre‐ and post‐intervention using commercial colorimetric kits. Statistical analysis was performed using paired *t*‐tests or Wilcoxon signed‐rank tests.

**Results:**

Vitamin D3 supplementation significantly increased serum 25(OH)D levels (20.80 ± 8.6 to 39.11 ± 12.26 ng/mL; *p* < 0.001). CHI3L1 concentration decreased by 21.7% (33.28 ± 8.9 to 26.05 ± 9.1 ng/mL; *p* < 0.001). oxidative stress was reduced, evidenced by lower TOS (1.55 ± 0.50 to 0.59 ± 0.23 mmol H_2_O_2_ equiv./L; *p* < 0.001) and MDA (0.08 ± 0.03 to 0.05 ± 0.026 nmol/mL; *p* < 0.001). Antioxidant capacity improved, as demonstrated by elevated TAC (0.622 ± 0.138 to 0.797 ± 0.15 mmol Fe^2+^/L; *p* < 0.001) and increased activities of SOD (10.5%; *p* < 0.001), CAT (19.5%; *p* < 0.001), and GPx (35.6%; *p* < 0.05). Significant inverse correlations were observed between serum 25(OH)D and CHI3L1 (*r* = −0.999, *p* < 0.001), TOS (*r* = −0.456, *p* = 0.0058), and MDA (*r* = −0.577, *p* < 0.001).

**Conclusion:**

Vitamin D3 supplementation was associated with reductions in CHI3L1 and oxidative stress markers, while suggesting enhancement of antioxidant capacity. This observed biomarker changes support vitamin D3 as a potential adjunct therapy targeting interconnected pathological pathways in MS.

## Abstract 2

In multiple sclerosis (MS) as a chronic autoimmune neurodegenerative disorder, 8‐week vitamin D3 supplementation was associated with reductions in the neuroinflammatory biomarker Chitinase‐3‐like protein 1 (CHI3L1) and oxidative stress markers, while suggesting enhancement of antioxidant capacity. Nevertheless, the observed biomarker changes support vitamin D3 as a potential adjunct therapy targeting interconnected pathological pathways in MS.

## Graphical abstract

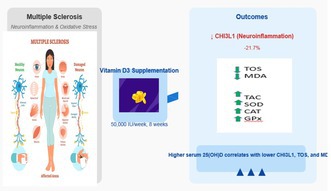

## 1 Introduction

Multiple sclerosis (MS) is a chronic, immune‐mediated, neurodegenerative disorder that primarily affects the central nervous system (CNS). It is characterized by demyelination, axonal injury, gliosis, and chronic inflammation, resulting in varied neurological symptoms including fatigue, visual disturbances, motor dysfunction, and cognitive impairment [1, 2, 3]. MS typically manifests in young adults (20–40 years) and shows a marked female predominance, with female‐to‐male ratios up to 3:1. Globally, an estimated 1.89 million individuals are affected by MS, with over 62 000 new cases diagnosed in 2021. The worldwide prevalence rate for MS is recorded at 23.9 cases per 100 000 population, a figure that has consistently increased over the past three decades [1, 4]. In Iran, the prevalence rate is notably high, with a significant impact on the population, particularly in regions with less sunlight exposure, potentially correlating with vitamin D deficiency [5].

The etiology of MS remains multifactorial and incompletely understood. Genetic susceptibility, epigenetic influences, and environmental exposures collectively contribute to its onset and progression. Among the recognized environmental risk factors, vitamin D deficiency, Epstein–Barr virus infection, and smoking have received considerable attention [6, 7]. Recent studies suggest that vitamin D deficiency may act synergistically with these risk factors, for example by enhancing immune susceptibility to Epstein–Barr virus or by failing to counteract smoking‐related oxidative stress, thereby amplifying MS risk. However, the precise mechanisms of these interactions remain under investigation. Notably, higher MS prevalence is observed in regions farther from the equator, suggesting a potential link with sunlight exposure and vitamin D synthesis [1, 8]. Oxidative stress and neuroinflammation are central to the pathogenesis of MS. Increased production of reactive oxygen species (ROS) and impaired antioxidant defenses contribute to demyelination and neuronal injury, particularly affecting oligodendrocytes and neurons due to their limited antioxidant capacity [9]. Excessive ROS and reactive nitrogen species (RNS) disrupt the blood–brain barrier (BBB), promote leukocyte infiltration, and exacerbate inflammatory and degenerative processes in the CNS [2, 10, 11]. CHI3L1 (Chitinase‐3‐like protein 1), also known as YKL‐40, is a secreted glycoprotein that plays a significant role in inflammation, tissue remodeling, and neurodegeneration. Despite lacking chitinase enzymatic activity, CHI3L1 binds chitin and other extracellular matrix components, acting as a signaling molecule through interactions with receptors such as RAGE, IL‐13Rα2, and syndecan‐1/αVβ3 [12, 13, 14, 15]. In the CNS, CHI3L1 is primarily expressed by activated astrocytes and microglia, particularly in active demyelinating lesions, and is considered a biomarker of glial activation and neuroinflammation [16, 17, 18]. In MS, elevated levels of CHI3L1 have been detected in both CSF and serum, correlating with disease activity, lesion burden, and progression. It is believed to contribute to neuroinflammatory cascades by modulating cytokine production and amplifying microglial and astrocyte responses [14, 15, 19]. Importantly, CHI3L1 expression is inducible by pro‐inflammatory cytokines such as IL‐1β, IL‐6, and TNF‐α. These pathways may be modulated by vitamin D3, although direct evidence in MS remains limited [20]. Current therapeutic strategies include disease‐modifying therapies (DMTs) such as interferons, glatiramer acetate, sphingosine‐1‐phosphate modulators, fumarates, and monoclonal antibodies, which can reduce relapse rates and slow disease progression but are associated with significant costs and potential adverse effects [21]. Vitamin D3, a fat‐soluble secosteroid hormone, has emerged as a critical neuroimmunomodulatory factor in MS. It is synthesized in the skin upon UVB exposure and activated through hepatic and renal hydroxylation. Its active form, 1,25‐dihydroxyvitamin D, acts via the vitamin D receptor (VDR) to regulate gene transcription in immune and neural cells. Vitamin D3 influences both innate and adaptive immune responses by promoting anti‐inflammatory cytokines (e.g., IL‐10) and suppressing pro‐inflammatory mediators (e.g., IL‐17, IFN‐γ). The presence of VDR in T cells, B cells, macrophages, and microglia further supports its potential role in MS pathophysiology [22, 23, 24, 25, 26]. Numerous studies have demonstrated an inverse association between serum 25(OH)D levels and MS incidence and disease activity. Vitamin D3 supplementation has been linked to reduced relapse rates, lower lesion burden on MRI, and improved immune regulation [27, 28]. Beyond its immunomodulatory roles, vitamin D3 also acts as a potent antioxidant, enhancing the expression of genes involved in redox balance and protecting neural tissues from oxidative damage [9, 29]. Nevertheless, evidence remains inconsistent; several studies and meta‐analyses have reported no significant association between vitamin D status and oxidative stress markers in MS, highlighting heterogeneity in patient responses and underscoring the need for further research. Several lines of evidence suggest that vitamin D may downregulate CHI3L1 expression and exert anti‐inflammatory effects in MS. Vitamin D3 has been shown to suppress pro‐inflammatory mediators in glial cells and enhance neuroprotective factors. It may also mitigate oxidative stress, indirectly influencing CHI3L1 through redox‐sensitive pathways [9, 30, 31]. However, direct evidence of vitamin D–mediated CHI3L1 downregulation in MS is limited, and alternative mechanisms such as NF‐κB modulation, Nrf2 pathway activation, or indirect metabolic effects cannot be excluded. Given the importance of oxidative stress and inflammation in MS pathology and the involvement of CHI3L1 in both processes, interventions targeting these pathways are of therapeutic interest. Antioxidant enzymes such as superoxide dismutase (SOD), catalase (CAT), and glutathione peroxidase (GPx) are essential in neutralizing ROS and maintaining cellular redox balance. Reduced activity of these enzymes has been reported in MS patients, supporting the hypothesis of compromised antioxidant defense [10, 32, 33]. Enhancing antioxidant capacity via vitamin D3 supplementation may offer neuroprotective benefits and modulate disease progression. Vitamin D3 has multifaceted roles in immune modulation, oxidative stress mitigation, and inflammation regulation. This study aims to evaluate its effects on serum CHI3L1 levels and oxidative stress parameters in patients with MS. Considering vitamin D3's multifaceted roles in immune modulation, oxidative stress mitigation, and inflammation regulation, this study aims to evaluate its effects on serum CHI3L1 and oxidative stress parameters in MS patients. By incorporating both supportive and conflicting evidence and acknowledging speculative aspects of the CHI3L1 hypothesis, we provide a balanced rationale for the present investigation.

## 2 Materials and Methods

### 2.1 Study Design

This study was conducted as a single‐arm, open‐label pre–post clinical trial between 2022 and 2023 at the Biochemistry Research Laboratory of Hamadan University of Medical Sciences and the Neurology Department of Besat Hospital, Hamadan, Iran. The trial did not include randomization, placebo control, or blinding, which are acknowledged as potential limitations. These limitations may introduce placebo effects, seasonal variation in vitamin D levels, and confounding factors such as disease‐modifying therapies (DMTs) that could influence the results. Despite these limitations, the study provides valuable insight into the effects of vitamin D3 supplementation on MS patients.

### 2.2 Participants

A total of 35 patients diagnosed with MS, aged between 18 and 65 years, were recruited from the Neurology Department of Besat Hospital. The diagnosis was confirmed by a specialist neurologist based on clinical and paraclinical criteria. Inclusion criteria were defined as the absence of vitamin D3 supplementation in the past 2 months and written informed consent. Exclusion criteria included current or recent use of other antioxidant supplements, irregular use of the prescribed vitamin D3 during the study period, acute infections, autoimmune or rheumatic diseases, diabetes mellitus, renal dysfunction, malabsorption syndromes, hypersensitivity to vitamin D3, and age outside the 18–65 range. Data on participants' ongoing DMTs, including interferons, glatiramer acetate, or oral agents, were recorded. Patients continued their prescribed DMTs during the study; no changes in the DMT regimen occurred during the 8‐week supplementation. While DMTs were not an exclusion criterion, treatment stability was ensured during the intervention period. A power calculation was performed prior to the study to justify the sample size (*n* = 35), ensuring sufficient statistical power to detect meaningful changes.

### 2.3 Intervention and Sample Collection

After obtaining baseline data, patients received 50 000 IU/week of oral vitamin D3 (Zahravi Pharmaceutical Co., Iran) for 8 weeks. Blood samples (5 mL) were collected from all participants both at baseline and at the end of the supplementation period. Samples were obtained in gel clot tubes, centrifuged to separate serum. The selected dose (50 000 IU/week) is consistent with regimens commonly used for short‐term correction of vitamin D deficiency in MS. Safety monitoring included clinical surveillance for hypercalcemia‐related symptoms and assessment of serum calcium at baseline and after the 8‐week supplementation. Serum aliquots were initially stored at −20°C for interim handling, then transferred to −80°C within 48 h for long‐term storage, in accordance with best practice guidelines for oxidative stress and enzyme activity assays. We acknowledge that if samples were held at −20°C for longer periods, this may represent a limitation.

### 2.4 Assessment of 25‐Hydroxyvitamin D

Serum 25(OH)D levels were measured using a competitive enzyme‐linked immunosorbent assay (ELISA) kit (Biokarpira, Iran), performed at the Besat Hospital diagnostic laboratory, Hamadan, Iran. This analyte was specifically targeted over the active form (1,25‐dihydroxyvitamin D) due to its approximately 1000‐fold higher serum concentration, making it the established clinical marker for assessing vitamin D3 status and deficiency. Microtiter wells were pre‐coated with anti‐vitamin D antibody. Serum samples were added, followed by an extraction buffer containing a Biotinylated Vitamin D analog (Biotin‐Ag). Endogenous 25(OH)D and the Biotin‐Ag competed for binding sites on the immobilized antibody. After washing, an Avidin‐Horseradish Peroxidase (Avidin‐HRP) conjugate was added, binding to the captured Biotin‐Ag to form an Avidin‐HRP‐Biotin complex. Following another wash step, a chromogenic substrate solution containing hydrogen peroxide (H_2_O_2_) and tetramethylbenzidine (TMB) was added, producing a blue color. The reaction was stopped with sulfuric acid, converting the color to yellow, with maximum absorbance measured at 450 nm using a microplate reader and expressed in ng/mL.

### 2.5 Assessment of Chitinase‐3‐Like Protein 1

Serum CHI3L1 concentration was determined using a biotin‐streptavidin based sandwich ELISA kit (ZellBio, Germany). Microtiter wells pre‐coated with monoclonal anti‐CHI3L1 antibody received 40 μL of serum sample. Subsequently, 10 μL of biotinylated anti‐CHI3L1 detection antibody and 50 μL of streptavidin‐HRP conjugate were added. Plates were incubated at 37°C for 60 min to form immune complexes. After aspiration, wells were washed 5 times with 300 μL of wash buffer. Then, 100 μL of chromogen substrate solution was added and incubated for 10–20 min at 37°C in the dark for color development. The reaction was stopped by adding 50 μL of stop solution, changing the color from blue to yellow. After a final 10‐min incubation, absorbance was measured at 450 nm using a microplate reader and expressed in ng/mL.

### 2.6 Oxidative Stress Markers

#### 2.6.1 Assessment of Total Antioxidant Capacity (TAC)

Serum TAC was assessed using the Ferric Reducing Ability of Plasma (FRAP) method with a commercial kit (Navand Salamat, Iran) as previously validated for clinical samples [34]. The assay relies on the single‐electron transfer reduction of ferric ions (Fe^3+^) to ferrous ions (Fe^2+^) by antioxidants present in the sample. This reduces the Fe^3+^‐Tripyridyltriazine (Fe^3+^‐TPTZ) complex to the intensely blue‐colored Fe^2+^‐TPTZ complex. Briefly, 5 μL of serum or standard was mixed with 250 μL of the pre‐prepared FRAP working reagent. After a 5‐min incubation at 37°C, absorbance was measured at 593 nm using a Mobi microplate reader. TAC values, proportional to the absorbance, are expressed as mmol Fe^2+^ equivalents per liter (mmol Fe^2+^/L).

#### 2.6.2 Assessment of Total Oxidant Status (TOS)

Serum TOS, representing the cumulative concentration of oxidants including reactive oxygen and nitrogen species, was measured colorimetrically using a kit (Navand Salamat, Iran). The assay is based on the oxidation of o‐dianisidine, catalyzed by oxidants present in the sample in the presence of ferric ions (Fe^3+^ from FeCl_3_). Fe^3+^ acts as an electron acceptor, being reduced to Fe^2+^ during the reaction. The oxidation of o‐dianisidine generates a colored product. In the procedure, 30 μL of serum or standard was combined with 200 μL of Reagent 1 and 10 μL of Reagent 2. After a 20‐min incubation at 37°C, absorbance was measured at 530 nm using a Mobi microplate reader. Absorbance is directly proportional to the total oxidant level. Results are expressed as mmol hydrogen peroxide equivalents per liter (mmol H_2_O_2_ Equiv./L).

#### 2.6.3 Assessment of Malondialdehyde (MDA)

Serum MDA, a marker of lipid peroxidation, was quantified using the thiobarbituric acid reactive substances (TBARS) method via an ELISA kit (Navand Salamat, Iran). MDA, generated during the peroxidation of polyunsaturated fatty acids, reacts with thiobarbituric acid (TBA) under high temperature and acidic conditions to form a pink chromophore. Briefly, 200 μL of serum or standard was mixed with 800 μL of the TBA‐containing working solution. The mixture was incubated at 95°C for 45 min in a water bath, then rapidly cooled on ice for 10 min. Samples were centrifuged at 3000 *g* for 15 min. Subsequently, 250 μL of the supernatant was transferred to microplate wells, and absorbance was measured at 550 nm using a microplate reader. Results were expressed as nmol/mL.

### 2.7 Antioxidant Enzyme Assessment

#### 2.7.1 Superoxide Dismutase (SOD) Activity Assay

Cytosolic (Cu/Zn‐SOD) activity was specifically measured in serum using a commercial kit (Navand Salamat, Iran) based on the inhibition of pyrogallol autoxidation. Serum samples were centrifuged at 10 000 *g* for 15 min at 4°C to isolate the cytosolic fraction. For the assay, 50 μL of serum supernatant was combined with 200 μL of Reagent 1 and 50 μL of Reagent 2 in microplate wells. Following a 5‐min incubation at 37°C, absorbance was measured at 405 nm using a Mobi microplate reader.

#### 2.7.2 Catalase (CAT) Activity Assay

Catalase activity was quantified using a commercial kit (Navand Salamat, Iran) measuring its peroxidatic function via formaldehyde generation. Serum was separated by centrifugation (2000 *g*, 15 min, 4°C). In the assay, 100 μL of diluted Assay Buffer and 30 μL of Reagent 1 were added to wells, followed by 20 μL of serum or formaldehyde standard. The reaction was initiated by adding 20 μL of Reagent 2 (containing methanol and H_2_O_2_). Samples were continuously shaken on a rotator at < 20°C for 20 min. The reaction was stopped by adding 30 μL of Reagent 3 (KOH). Subsequently, 30 μL of Reagent 4 (containing chromogen) was added, and samples were shaken again at < 20°C for 10 min. Finally, 10 μL of Reagent 5 was added, and absorbance was measured at 550 nm (±20 nm) using a microplate reader. Results are expressed as μmol of H_2_O_2_ decomposed per minute per liter (μmol/min/L).

#### 2.7.3 Glutathione Peroxidase (GPx) Activity Assay

GPx activity was assessed using a coupled enzyme assay kit (Navand Salamat, Iran) based on the oxidation of NADPH at 340 nm. Cumene hydroperoxide serves as the substrate. Reduced glutathione (GSH) is oxidized to glutathione disulfide (GSSG) by GPx, utilizing cumene hydroperoxide. GSSG is then continuously reduced back to GSH by glutathione reductase (GR), consuming NADPH. The rate of NADPH oxidation is directly proportional to GPx activity. Briefly, 50 μL of serum was added to wells followed by 40 μL of Reagent 1. After a 15‐min incubation, the reaction was initiated by adding 10 μL of Reagent 2. Absorbance at 340 nm was measured immediately and again after a 10‐min incubation. Results are expressed as nmol of NADPH oxidized per minute per mL (nmol/min/mL).

All spectrophotometric measurements were performed using microplate readers (Tecan Sunrise, Austria; Mobi, South Korea).

### 2.8 Statistical Analysis

Normality of distribution was evaluated using the Kolmogorov–Smirnov and Shapiro–Wilk tests. For normally distributed variables (vitamin D, TAC, TOS, MDA, GPx, CHI3L1), paired *t*‐tests were used to compare pre‐ and posttreatment values. For non‐normally distributed variables (SOD, CAT), the Wilcoxon signed‐rank test was employed. The effect size for paired *t*‐tests was calculated using eta squared (*η* ^2^), and for the Wilcoxon signed‐rank test, effect sizes were calculated using Spearman's rank correlation coefficient (*r*_s_) which provides the proportion of variance explained by the intervention. Data are presented as mean ± SD and visualized using mean ± SEM in plots. Statistical analyses were performed using GraphPad Prism (version 8.0). Given the number of biomarkers analyzed, *p*‐values were adjusted for multiple comparisons using the Bonferroni correction to reduce the risk of type I error.

## 3 Results

### 3.1 Demographic Characteristics

The study cohort comprised 35 patients with a confirmed diagnosis of MS. All participants were female (100%). The age distribution ranged from 30 to 56 years, with a mean age of 42.23 ± 7.45 years (mean ± standard deviation, SD). Demographic characteristics are summarized in Table 1.

**TABLE 1:** Demographic characteristics of the study cohort (*n* = 35).

Characteristic	Value	Description
Sex	Female: 35 (100%)	*n* (%)
Age (years)	45.7 ± 6.8	Mean ± SD
Age range	30–56	Min–max
Disease	Multiple sclerosis	All participants

### 3.2 25‐Hydroxyvitamin D Level

**FIGURE 1:**
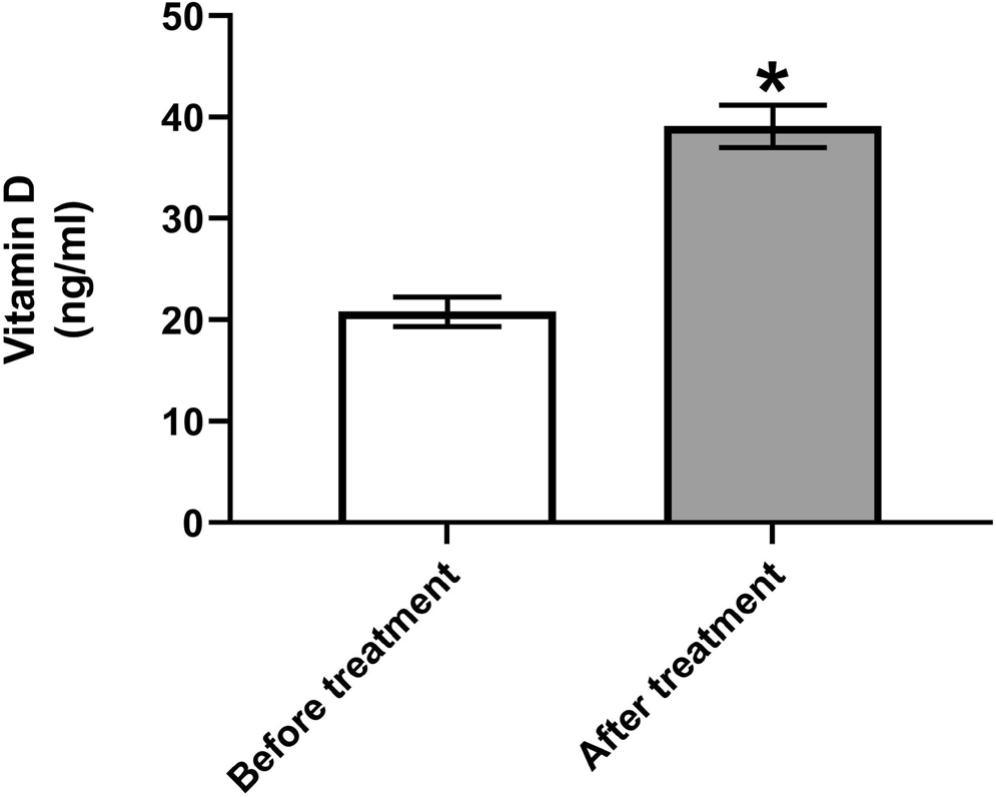
Serum 25‐hydroxyvitamin D concentrations before and after 8 weeks of vitamin D supplementation (*n* = 35). Data presented as mean ± SEM. *p < 0.05 (paired *t*‐test).

Vitamin D3 supplementation significantly increased serum 25‐hydroxyvitamin D concentrations in MS patients after 8 weeks of intervention (*p* < 0.001). Baseline levels were 20.80 ± 8.6 ng/mL (mean ± SD), rising to 39.11 ± 12.26 ng/mL post‐treatment (Figure 1). Statistical analysis using a paired *t*‐test revealed a robust treatment effect (*t* (34) = 12.63, *p* < 0.001), with a mean increase of 18.31 ng/mL (95% CI: 15.37–21.26). The effect size was substantial (partial *η* ^2^ = 0.824), indicating that vitamin D supplementation explained 82.4% of the variance in serum levels. The strong pretreatment–posttreatment correlation (*r* = 0.715, *p* < 0.001) confirmed effective within‐subject pairing.

### 3.3 Oxidative Stress Markers

#### 3.3.1 Total Antioxidant Capacity

**FIGURE 2:**
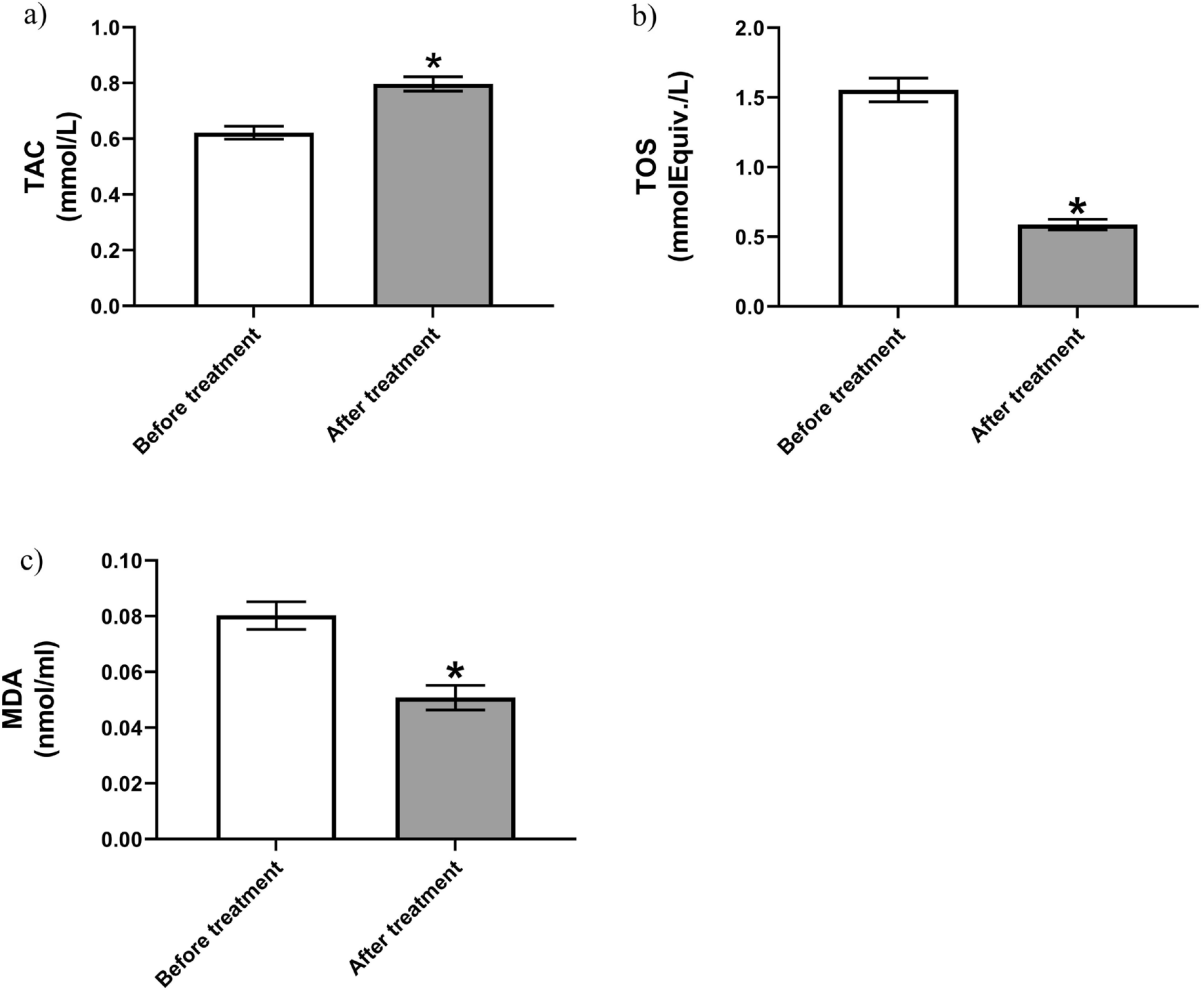
Oxidative stress markers included: serum total antioxidant capacity (a), serum total oxidant status (b), serum malondialdehyde (c) before and after 8 weeks of vitamin D supplementation (*n* = 35). Data presented as mean ± SEM. *p < 0.05 (paired *t*‐test).

Vitamin D supplementation significantly increased serum TAC in MS patients (*p* < 0.001). Baseline levels were 0.622 ± 0.138 mmol Fe^2+^/L (mean ± SD), rising to 0.797 ± 0.15 mmol Fe^2+^/L post‐treatment. Statistical analysis revealed a robust effect (*t* (34) = 11.95, *p* < 0.001), with a mean increase of 0.175 mmol Fe^2+^/L (95% CI: 0.146–0.205). The large effect size (*η* ^2^ = 0.808) and strong pretreatment–posttreatment correlation (*r* = 0.826, *p* < 0.001) confirmed both clinical significance and effective pairing (Figure 2a).

#### 3.3.2 Total Oxidant Status (TOS)

A significant reduction in serum TOS was observed post‐supplementation (*p* < 0.001). Levels decreased from 1.55 ± 0.50 mmol H_2_O_2_ equiv./L to 0.59 ± 2.3 mmol H_2_O_2_ equiv./L (mean difference: −0.966, 95% CI: −1.120 to −0.812; *t* (34) = 12.73). The large effect size (*η* ^2^ = 0.827) and significant correlation (*r* = 0.456, *p* = 0.0058) indicate vitamin D effectively reduces oxidative burden (Figure 2b).

#### 3.3.3 Malondialdehyde

Vitamin D supplementation significantly reduced lipid peroxidation (*p* < 0.001). MDA levels decreased from 0.08 ± 0.03 nmol/L to 0.05 ± 0.026 nmol/L (mean difference: −0.029, 95% CI: −0.038 to −0.021; *t* (34) = 6.80). The moderate effect size (*η* ^2^ = 0.576) and strong correlation (*r* = 0.577, *p* < 0.001) confirm reduced oxidative damage (Figure 2c).

### 3.4 Antioxidant Enzyme Activities

#### 3.4.1 Superoxide Dismutase

**FIGURE 3:**
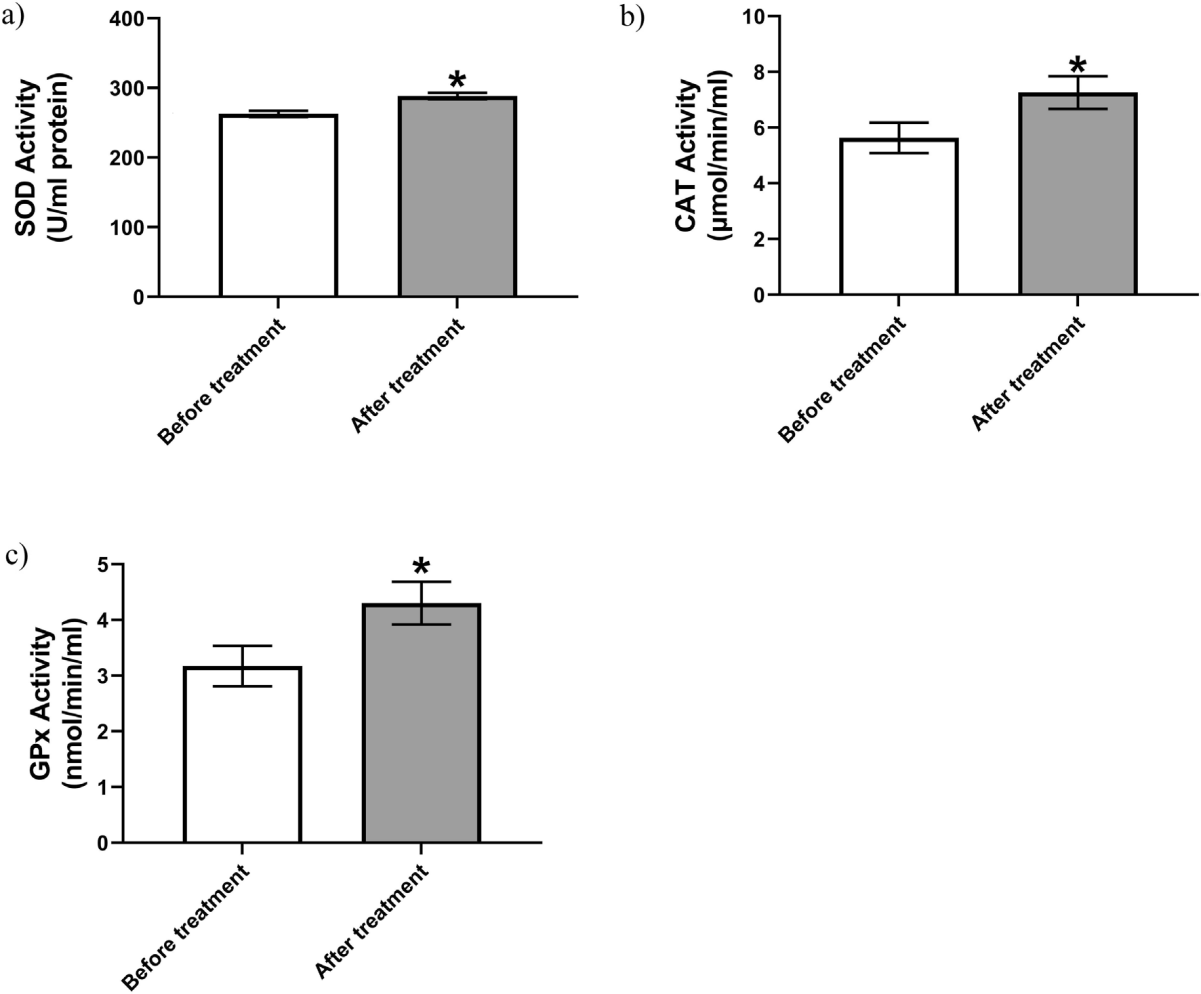
Antioxidant enzymes included: serum superoxide dismutase (a), serum catalase (b), serum glutathione peroxidase (c) activity before and after 8 weeks of vitamin D supplementation (*n* = 35). Data presented as mean ± SEM. *p < 0.05 (Wilcoxon signed‐rank test was used for SOD and CAT, and the paired *t*‐test for GPx).

SOD activity increased significantly after supplementation (*p* < 0.001, Wilcoxon test). Median activity rose by 26.83 U/mL (pre: 254.1 ± 26.20 U/mL; post: 280.7 ± 27.5 U/mL). The near‐perfect correlation (*r*_s_ = 0.999, *p* < 0.001) indicates consistent enhancement of superoxide radical detoxification (Figure 3a).

#### 3.4.2 Catalase

A significant increase in CAT activity was observed (*p* < 0.001, Wilcoxon test). Median activity increased by 1.14 μmol/min/L (pre: 3.2 ± 5.27 μmol/min/L; post: 3.4 ± 6.3 μmol/min/L). The moderate correlation (*r*_s_ = 0.457, *p* = 0.0058) suggests improved hydrogen peroxide metabolism (Figure 3b).

#### 3.4.3 Glutathione Peroxidase

GPx activity showed a modest but significant increase (*p* < 0.05). Levels rose from 2.14 ± 3.17 nmol/min/mL to 2.26 ± 4.3 nmol/min/mL (mean difference: 1.128, 95% CI: 0.165–2.091; *t* (34) = 2.38). The small effect size (*η* ^2^ = 0.143) indicates subtle enhancement of glutathione utilization (Figure 3c).

### 3.5 Serum CHI3L1 (YKL‐40) Levels

**FIGURE 4:**
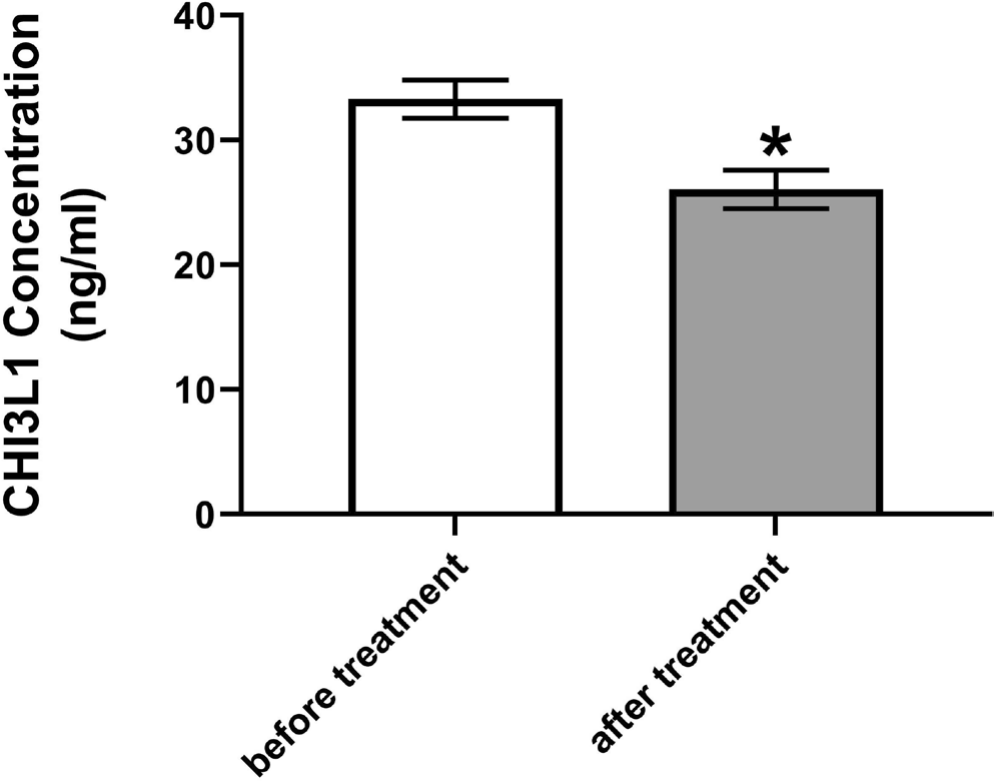
Serum CHI3L1 concentrations before and after 8 weeks of vitamin D supplementation (*n* = 35). Data presented as mean ± SEM. *p < 0.05 (paired *t*‐test).

The pro‐inflammatory glycoprotein CHI3L1 exhibited a significant decrease in serum concentration following vitamin D supplementation (*p* < 0.001). Baseline levels averaged 33.28 ± 8.9 ng/mL (mean ± SD), declining to 26.05 ± 9.1 ng/mL post‐treatment (Figure 4), with a mean reduction of 7.23 ng/mL (95% CI: −7.35 to −7.11; *t* (34) =120.0, *p* < 0.001). Suggesting a potential modulatory effect of vitamin D on this neuroinflammatory biomarker. The exceptional effect size (*η* ^2^ = 0.998) and near‐perfect correlation (*r* = 0.999, *p* < 0.001) demonstrate potent anti‐inflammatory effects. Despite the large effect sizes observed across several outcomes (e.g., *η* ^2^ = 0.824 for vitamin D, *η* ^2^ = 0.998 for CHI3L1), the absence of a control group prevents definitive causal attribution. These strong statistical signals may in part reflect natural variability or regression to the mean, underscoring the need for randomized controlled trials to confirm these findings.

Correlation analyses demonstrated robust inverse associations between serum 25(OH)D and CHI3L1 (*r* = −0.999, *p* < 0.001), TOS (*r* = −0.456, *p* = 0.0058), and MDA (*r* = −0.577, *p* < 0.001). These relationships, visualized in scatter plots provided in the Appendices S1 and S2, further support the link between vitamin D status, inflammation, and oxidative stress.

Finally, it must be emphasized that all participants were female, which limits the ability to extrapolate findings to male MS patients. Moreover, the modest sample size restricted subgroup analyses by age or disease duration. These constraints highlight the importance of future studies with larger, sex‐balanced cohorts to validate and extend the present observations.

## 4 Discussion

MS is a chronic autoimmune disorder of the CNS characterized by inflammation, demyelination, and axonal degeneration. Growing evidence implicates vitamin D deficiency as a significant environmental risk factor for MS development and progression, while also highlighting its potential immunomodulatory and neuroprotective roles [35, 36, 37, 38, 39]. This study investigated the effects of vitamin D supplementation on key inflammatory and oxidative stress biomarkers in MS patients, revealing a marked decrease in the inflammatory protein CHI3L1 and oxidative stress markers (MDA, TOS), alongside increases in TAC and enzymatic activity (SOD, CAT, GPx) following an 8‐week intervention. These findings underscore the pleiotropic effects of vitamin D in modulating pathways central to MS pathophysiology. It is important to emphasize, however, that the present findings are restricted to biomarker modulation. No clinical outcomes such as relapse frequency, Expanded Disability Status Scale (EDSS), or MRI lesion activity were measured. Therefore, while the observed biomarker changes suggest potential biological effects of vitamin D, they do not establish therapeutic benefit. VDRs are ubiquitously expressed throughout human tissues, including critical brain regions such as the hippocampus, substantia nigra, hypothalamus, thalamus, and neocortex [35, 36]. Within the CNS, vitamin D influences crucial processes like neuronal differentiation, migration, synaptogenesis, neurogenesis, and neuroprotection [37]. Epidemiological studies consistently report an inverse association between serum 25‐hydroxyvitamin D [25(OH)D] levels and MS risk, prevalence, and disease activity [38, 39, 40, 41, 42]. Populations with greater sun exposure exhibit lower MS incidence, implicating vitamin D's preventive potential [40]. Clinically, lower serum 25(OH)D levels correlate with increased T2 lesion burden in relapsing–remitting MS (RRMS) patients. Furthermore, randomized controlled trials demonstrate that cholecalciferol supplementation significantly reduces new active T1 lesions and T1 hypointense lesion volume in RRMS, suggesting a disease‐modifying effect [41]. Lower serum 25(OH)D levels are also observed during MS relapses compared to remission phases, supporting its immunomodulatory role in autoimmune dysregulation [42, 43]. A key finding of this study is the noticeable decline in serum CHI3L1 levels following vitamin D supplementation. CHI3L1, primarily secreted by activated astrocytes and microglia within less inflammatory CNS lesions, is a biomarker increasingly recognized for its prognostic value in MS. [12, 44, 45] Elevated serum CHI3L1 levels predict faster conversion from clinically isolated syndrome (CIS) to RRMS [44, 46] and are significantly higher in CIS and RRMS patients compared to healthy controls [47]. Our results demonstrate that while vitamin D supplementation appears effectively to lower serum CHI3L1 levels, it is important to note that the study did not control for potential confounding factors, such as concurrent DMTs for MS. Therefore, while these findings suggest a potential effect of vitamin D, causal conclusions cannot be definitively made without further studies including a control group. This aligns with emerging clinical and preclinical evidence. Omidian et al. reported significant CHI3L1 reduction after vitamin D supplementation in type 2 diabetic patients with vitamin D deficiency [48]. Similarly, Kocabas et al. found vitamin D effectively suppressed serum CHI3L1 in a hypercholesterolemic mouse model [49]. These collective findings suggest vitamin D may exert part of its anti‐inflammatory effect in MS by downregulating CHI3L1, potentially interrupting chronic inflammation. The mechanisms linking vitamin D and CHI3L1 likely involve cytokine modulation. CHI3L1 production is stimulated by various pro‐inflammatory cytokines, including TNF‐α, IL‐6, IL‐13, and IL‐1β, and by Th1 cytokines like IFN‐γ in human macrophages, while being suppressed by Th2 cytokines like IL‐4 [50, 51, 52, 53]. Vitamin D profoundly impacts cytokine profiles, inhibiting IL‐12, IL‐2, IFN‐γ, and IL‐17 production [54, 55, 56], while promoting anti‐inflammatory IL‐10 [57]. This shift away from pro‐inflammatory stimuli likely contributes to reduced CHI3L1 expression. Furthermore, vitamin D may directly or indirectly suppress CHI3L1 by inhibiting inflammatory mediators like sICAM1, sVCAM1, and IL‐6, or through metabolic pathways intersecting with VDR signaling [58, 59]. Oxidative stress plays a dominant role in MS pathogenesis, contributing significantly to cellular damage. An imbalance between ROS production and antioxidant defenses leads to lipid peroxidation, protein oxidation, and DNA damage [60, 61, 62]. The CNS is particularly vulnerable due to its high oxygen demand and relatively low antioxidant defenses [62]. Elevated markers of oxidative damage, including MDA, and reduced TAC are well‐documented in MS patients [60, 61]. Our results demonstrate that vitamin D supplementation significantly ameliorates oxidative stress in MS: reducing serum MDA (a marker of lipid peroxidation) and TOS (total oxidant status), while increasing TAC (total antioxidant capacity) and the activity of key antioxidant enzymes SOD (superoxide dismutase), CAT (catalase), and GPx (glutathione peroxidase). This aligns with meta‐analyses indicating vitamin D supplementation can significantly increase serum TAC and reduce MDA [29, 63], although some studies report variations in specific markers [64]. Hwaidi et al. also observed an inverse correlation between serum vitamin D and TOS in rheumatoid arthritis [65]. Our findings support previous evidence that vitamin D enhances antioxidant defenses. In the present study, these effects were reflected by increased TAC and enzymatic activity (SOD, CAT, GPx), suggesting that vitamin D's multifaceted antioxidant mechanisms—such as Nrf2 activation and suppression of NADPH oxidase—may underlie the observed biomarker improvements. Primarily, via activation of the VDR, it enhances cellular antioxidant defenses by upregulating the expression and activity of key antioxidant enzymes, including SOD, CAT, and GPx [60, 66, 67, 68]. Furthermore, vitamin D acts as a potent activator of the Nrf2‐KEAP1 pathway [69, 70, 71]. Under oxidative stress, this activation promotes the dissociation of nuclear factor erythroid 2‐related factor 2 (Nrf2) from its inhibitor KEAP1, enabling Nrf2 translocation to the nucleus. Here, it binds to the Antioxidant Response Element (ARE), driving the transcription of a wide array of antioxidant genes, including those for SOD, CAT, GPx, and glutathione synthesis enzymes [72, 73]. The importance of this pathway is underscored by findings that Nrf2 deficiency exacerbates experimental autoimmune encephalomyelitis (EAE), while its activation reduces clinical severity [71]. Vitamin D also combats oxidative stress by directly inhibiting major ROS sources, notably through VDR‐mediated transcriptional downregulation of the NADPH oxidase complex (Nox2) [70, 74]. Finally, vitamin D contributes indirectly to reducing oxidative burden by suppressing pro‐inflammatory cytokines such as TNF‐α, IL‐1, IL‐6, and IL‐17 [30, 57, 75]. This reduction in inflammation minimizes the activation of immune cells and their associated oxidative burst, a significant contributor to tissue oxidative damage [60]. In MS, inflammation and oxidative stress form an inextricably linked and self‐perpetuating pathogenic cycle [76, 77, 78]. Oxidative stress acts as a key instigator, activating transcription factors such as nuclear factor kappa B (NF‐κB) and AP‐1. This activation drives the increased production of pro‐inflammatory cytokines (e.g., TNF‐α, IL‐6) and proteins, prominently including CHI3L1 [79]. These inflammatory mediators subsequently stimulate immune cells to generate further ROS, thereby amplifying oxidative stress [76, 77, 78]. Our findings, supported by existing literature, position CHI3L1 as a critical nexus within this damaging interplay. CHI3L1 levels rise significantly under conditions of both oxidative stress and chronic inflammation [80, 81, 82], and we observed a direct positive correlation between elevated TOS/MDA (markers of oxidative burden) and increased CHI3L1. Crucially, elevated CHI3L1 itself can activate pro‐inflammatory (NF‐κB) and stress‐responsive (Mitogen‐Activated Protein Kinase MAPK) signaling pathways, further fueling both inflammation and oxidative stress [82, 83]. Furthermore, a high oxidative burden (reflected by high TOS/MDA and low TAC) consumes and potentially depletes key antioxidant enzymes like SOD, CAT, and GPx, diminishing their protective activity [80]. However, these associations may also be subject to reverse causality or confounding by disease activity and progression, as higher CHI3L1 levels may reflect underlying MS severity rather than a direct effect of vitamin D. Vitamin D emerges as a uniquely positioned therapeutic agent within this context. Its demonstrated ability to concurrently reduce levels of CHI3L1, TOS, and MDA while enhancing TAC and the activity of SOD, CAT, and GPx equips it to disrupt this detrimental cycle at multiple critical points. This study provides compelling clinical evidence that vitamin D supplementation exerts significant anti‐inflammatory and antioxidant effects in MS patients, as evidenced by the reduction in serum CHI3L1, MDA, and TOS, and the increase in TAC, SOD, CAT, and GPx activity following an 8‐week intervention. These findings reinforce the potential of vitamin D as an adjunct therapy in MS management. The observed reduction in CHI3L1 is particularly noteworthy, suggesting a novel mechanism by which vitamin D may modulate neuroinflammation. Given CHI3L1's established role as a prognostic biomarker for disease conversion and activity [12, 44], its downregulation by vitamin D could have significant clinical implications. The concurrent amelioration of oxidative stress markers underscores vitamin D's capacity to target two fundamental, interconnected pathological processes in MS. This study provides evidence of beneficial biomarker modulation; however, several key limitations highlight important avenues for future research. Most critically, the single‐group, pre–post design precludes any causal inference. Without a placebo‐controlled comparator, we cannot exclude the possibility that observed changes were due to natural fluctuations, regression to the mean, or unmeasured confounding factors. Thus, the current findings should be interpreted as hypothesis‐generating rather than confirmatory. First, while promising biomarker changes were observed, larger longitudinal studies are essential to determine if these translate into tangible clinical benefits, such as reduced relapse rates, slowed disability progression, or improved MRI outcomes. Related to clinical translation, the optimal dose and duration of vitamin D supplementation required to maximize its anti‐inflammatory and antioxidant effects in MS patients remain to be fully defined. Furthermore, deeper mechanistic investigation is needed to elucidate how vitamin D signaling precisely leads to CHI3L1 downregulation in vivo within the human central nervous system, clarifying the relative contributions of direct VDR‐mediated actions versus indirect effects through cytokine modulation. Proposed mechanisms such as Nrf2 activation are derived primarily from preclinical or non‐MS studies, and evidence in MS patients remains inconsistent, particularly regarding vitamin D's effects on oxidative stress markers. These discrepancies highlight the need for direct mechanistic studies in the MS population. Additionally, while preclinical evidence supports vitamin D's activation of the Nrf2 pathway [71], its specific role and modulation by vitamin D in human MS CNS tissue require direct confirmation. Finally, the study population consisted exclusively of women, which restricts the generalizability of the findings to male patients. This was not an intentional choice but rather a consequence of the referral patterns during the study period. In addition, the modest sample size precluded subgroup analyses by age or disease duration. The study design, being a single‐arm trial without a control group, introduces the potential for bias and confounding factors. This limits the ability to draw definitive causal conclusions. Future studies with larger, sex‐balanced cohorts are therefore essential to validate and extend these observations.

## 5 Conclusion

Vitamin D deficiency is a modifiable risk factor in MS. In this study, vitamin D supplementation was associated with reductions in key inflammatory (CHI3L1) and oxidative stress (MDA, TOS) biomarkers, along with enhanced antioxidant defenses (TAC, SOD, CAT, GPx). These findings point to the potential role of vitamin D in modulating interconnected immune and oxidative pathways relevant to MS pathophysiology. However, given the single‐group pre–post design, relatively small, all‐female cohort, and lack of a placebo control, these results should be interpreted with caution and considered hypothesis‐generating rather than confirmatory. While the biomarker improvements are encouraging, clinical outcomes such as relapse rates, EDSS, or MRI lesion activity were not measured in this study. Further well‐designed randomized controlled trials with larger, more diverse populations and clinical outcome measures are warranted to determine whether these biological effects translate into meaningful therapeutic benefits in MS management.

## Author Contributions

Conceptualization, methodology: S.A., M.M., G.S., J.K., and I.K. Data analysis. S.A. Writing – original draft: S.A., G.S., J.K., and I.K. Review and editing: S.A., G.S., J.K. and I.K. Supervision: G.S.

## Funding

This research was funded by Hamadan University of Medical Sciences (Project No: 140206144762).

## Ethics Statement

This experiment was approved by the Ethics Committee of Hamadan University of Medical Sciences (ethics committee code: IR.UMSHA.REC.1401.984). This clinical trial study is also registered in the Iranian Registry of Clinical Trials (IRCT ID: IRCT20120215009014N473) and the trial protocol can be accessed on the IRCT website.

## Conflicts of Interest

The authors declare no conflicts of interest.

## Supporting information


**Appendix S1:** Supporting Information.

**Appendix S2:** Supporting Information.

## Data Availability

The raw data are available in Appendices S1 and S2.
